# Cardiopulmonary bypass time is an independent risk factor for acute kidney injury in emergent thoracic aortic surgery: a retrospective cohort study

**DOI:** 10.1186/s13019-019-0907-x

**Published:** 2019-05-07

**Authors:** Shijun Xu, Jie Liu, Lei Li, Zining Wu, Jiachen Li, Yongmin Liu, Junming Zhu, Lizhong Sun, Xinliang Guan, Ming Gong, Hongjia Zhang

**Affiliations:** 1 Department of Cardiac Surgery, Beijing Aortic Disease Center, Beijing Anzhen Hospital, Capital Medical University, Beijing Institute of Heart Lung and Blood Vessel Diseases, Beijing Lab for Cardiovascular Precision Medicine, and Beijing Engineering Research Center of Vascular Prostheses, No.2 Anzhen Street, Beijing, 100029 China; 2 Department of Vascular and Endovascular Surgery, Chinese PLA General Hospital, Beijing, 100853 China

**Keywords:** Acute kidney injury, Aortic dissection, Cardiopulmonary bypass, Risk factor, Thoracic aortic surgery

## Abstract

**Background:**

Thoracic aortic surgery and cardiopulmonary bypass are both associated with development of postoperative acute kidney injury. In this study, we undertook to investigate the relationship between cardiopulmonary bypass time and postoperative acute kidney injury in patients undergoing thoracic aortic surgery for acute DeBakey Type I aortic dissection.

**Methods:**

All patients receiving thoracic aortic surgery for acute DeBakey Type I aortic dissection in Beijing Anzhen hospital from December 2015 to April 2017 were included. Cardiopulmonary bypass time was recorded during surgery. Acute kidney injury was defined based on the Kidney Disease Improving Global Outcomes criteria. A total of 115 consecutive patients were eventually analyzed.

**Results:**

The overall incidence of acute kidney injury was 53.0% (*n* = 61). The average age was 47.8 ± 10.7 years; 74.8% were male. Mean cardiopulmonary bypass time was 211 ± 56 min. In-hospital mortality was 7.8%. Multivariate logistic regression revealed that cardiopulmonary bypass time was independently associated with the occurrence of postoperative acute kidney injury after adjust confounding factors (odds ratio = 1.171; 95% confidence interval: 1.002–1.368; *P* = 0.047).

**Conclusions:**

Cardiopulmonary bypass time is independently associated with an increased hazard of acute kidney injury after thoracic aortic surgery for acute DeBakey Type I aortic dissection. Further understanding of the mechanism of this association is crucial to the design of preventative strategies.

**Electronic supplementary material:**

The online version of this article (10.1186/s13019-019-0907-x) contains supplementary material, which is available to authorized users.

## Introduction

Acute kidney injury (AKI) is frequent as a serious complication following operation for acute DeBakey Type I aortic dissection (ADTIAD). But, the reported incidence of AKI following operation for ADTIAD varies extensively (20 to 67%), because the definition of AKI is different among studies, which is mildly higher than the incidences following other cardiac operations [8, 34, 36, 38, 40]. Furthermore, 2 to 8% of patients need renal replacement therapy (RRT) following aortic surgery [1, 2, 9, 10, 19, 20], which is related to an elevated short-term death rate of up to 64% [6, 10, 19, 20]. Previous studies had found that even mild AKI following cardiothoracic surgery would aggravate short-term outcomes such as 30- or 90-day mortality, morbidity, and cost [9, 21, 29]. As no efficient therapy for AKI is available currently [35], identifying risk factors and preventing AKI following cardiothoracic surgery are necessary parts of improving outcomes [7].

Cardiopulmonary bypass (CPB) is necessary to facilitate surgical correction of ADTIAD. However, it can result in significant inflammation and oxidant stress response which contribute to multi-organ dysfunction. Studies examining the association between CPB time and postoperative AKI in patients undergoing thoracic aortic surgery for ADTIAD have not been extensive.

For these reasons, we conducted a retrospective cohort study to investigate the relationship between CPB time and AKI in patients who underwent thoracic aortic procedure for ADTIAD using a multivariate logistic regression model containing all known associated major perioperative predictors. Our hypothesis was that the risk of AKI would increase as CPB time increased.

## Methods

### Participants

A retrospective cohort study was conducted at the Beijing Anzhen hospital from December 2015 to April 2017 in China, Beijing. This study was approved by the ethics committees of this hospital and conducted following the rules of Good Clinical Practice and principles of the Declaration of Helsinki. Individual consent was waived owing to the retrospective study. All patients who underwent aortic total arch replacement (TAR) combined with a frozen elephant trunk (FET) implant for ADTIAD in this timeframe were included. All operations were performed by the identical surgery team.

### Data collection

Trained staff collected detailed data from recruited patients from the electronic medical records at our medical center. The baseline characteristics collected for each patient involved age, gender, height, weight, body mass index (BMI, calculated based on height and weight recorded by the nurse on the day of hospital admission), drinking history, smoking history; Comorbidities: diabetes mellitus, hypertension, previous cerebrovascular disease, left ventricular ejection fraction (LVEF), coronary artery disease, preoperative hemoglobin, hematocrit, preoperative serum creatinine (sCr), preoperative blood urea nitrogen (BUN); eGFR (estimated glomerular filtration rate, calculated based on Epidemiology Collaboration equation), hemopericardium, renal artery dissection or occlusion, Penn class (Class Aa and Non class Aa), kidney malperfusion, acute myocardial infarction (AMI), preoperative shock; Intraoperative data: intraoperative transfusion of packed red blood cells (PRBCs), CPB time, aortic cross clamp time, circulatory arrest time, rectal temperature at circulatory arrest, nasopharyngeal temperature at circulatory arrest, the type of surgery (Bentall+TAR+FET or ascending aorta replacement+TAR+FET), combined with coronary artery bypass grafting (CABG) and combined with ascending aorta to femoral artery bypass surgery (aortic bypass surgery); Postoperative data: reoperation for bleeding, postoperative dialysis, length of intensive care unit (ICU) stay, length of time in hospital, sepsis, in-hospital death. It should be noted that in our study cohort, there was no patient underwent coronary angiography (CAG) within the 24 h before surgery and no patient received aprotinin or underwent statin therapy. Primary indications for postoperative dialysis were volume overload, uremia, anuria, and significant biochemical abnormalities.

The presence or absence of malperfusion was based on the Penn classification which was established and subsequent validated in the last decade [31]. Preoperative shock was defined as a systolic blood pressure < 90 mmHg [11]. Patients with ST elevation on a 12-lead electrocardiogram associated with wall hypokinesis at the corresponding region on echocardiography were considered to have AMI [33]. Renal malperfusion was diagnosed as at least one renal artery dissection with creatinine rise above 50% of the normal upper limit [11].

### Outcome variables

The primary endpoint event was AKI after thoracic aortic surgery for ADTIAD. Several classification criterions were established to access the postoperative AKI. Recently, KDIGO put forward a new range of guidelines for the classification of postoperative AKI based on the 2 previous classifications, RIFLE and AKIN [15, 24]. For the purpose of this study, postoperative AKI was diagnosed based on the KDIGO criteria: increase in sCr ≥ 0.3 mg/dL (in 48 h) or 1.5 times or greater by baseline (in 7 days).

### Assessment of covariates

Age, gender, diabetes mellitus, hypertension, preoperative hemoglobin, hematocrit, preoperative sCr, preoperative BUN, eGFR, renal artery dissection or occlusion, Penn class, kidney malperfusion, AMI, preoperative shock, CPB time, aortic cross clamp time, circulatory arrest time, nasopharyngeal temperature at circulatory arrest, rectal temperature at circulatory arrest, reoperation for bleeding were recorded in all participants. Preoperative sCr, BUN, hemoglobin, hematocrit was recorded based on the results of the initial laboratory test after admission before surgery.

### Surgical technique

Patients underwent median sternotomy and CPB. Briefly, the procedure is performed with right axillary artery cannulation for CPB and antegrade cerebral perfusion [5–15 mL/(kg•min)] under moderate hypothermic circulatory arrest (HCA). CPB was performed after systemic heparinization [300 U/kg and maintaining an activated clotting time (ACT) longer than 480 s]. Temperature-adjusted flow rates were 2.5 L/(min·m^2^) at the time of CPB. The mean arterial pressure was commonly maintained between 50 and 70 mmHg. After CPB was established, cooling was initiated. After clamping of the ascending aorta, cardiac arrest was accomplished with cold cardioplegic solution. Whether to perform an aortic valve replacement depended on the condition of the aortic valve. If the classification of aortic regurgitation was moderate or severe, we preferred to perform Bentall procedure (aortic valve replacement combined with ascending aorta replacement). If there was only mild regurgitation, we preferred to perform ascending aorta replacement only. All patients underwent TAR with FET. The method has been described in detail by our research team [26, 37]. An intraoperative stent-graft (MicroPort Medical Company Limited, Shanghai, China) and a four branched prosthetic graft (Maquet Cardiovascular, Wayne, NJ) were employed in this implantation. In brief, cannulation of the right axillary artery was used for CPB and unilateral selective cerebral perfusion (SCP). The distal aorta was transected circumferentially between the origin of the left common carotid artery and the origin of the left subclavian artery. The stent was implanted into the distal aorta. The distal aorta incorporating the stented elephant trunk was firmly attached to the distal end of the four-branched prosthetic graft using the “open” aortic method. Antegrade systemic perfusion was reestablished through the perfusion limb of the four-branched prosthetic graft. The anastomosis to the left common carotid artery was carried out first. After the anastomosis was completed, CPB was gradually returned to normal flow, SCP was discontinued, and rewarming was started. The anastomosis to the left subclavian artery, the innominate artery, and the proximal anastomosis were completed. If the blood pressure of the upper and lower limbs differed significantly and the signs and symptoms of lower limbs ischemia were presented, the ascending aorta to femoral artery bypass surgery was performed.

### Sample size

After excluding the 22 patients, a total of 115 consecutive patients underwent TAR combined with a FET implant for ADTIAD were included in the final analysis.

### Statistical analysis

Continuous variables were provided as median (quartile) or mean ± standard deviation (SD), in the light of the data dispersion. Categorical variables were expressed as percentages (%). The t-tests were used to compare if the continuous variables followed normal distribution, and if the variables were skewed distribution, non-parametric Mann–Whitney U tests were applied. The chi-square test was applied to compare with categorical variates. Logistic regression analysis was applied to identify the predictors of postoperative AKI. Multiple logistic regression analysis was used to evaluate the association between CPB time and postoperative AKI. We constructed four models: (I) adjusted for none; (II) adjusted for demographics: age; gender; (III) adjusted for age; gender; BMI; smoking history; aortic cross clamp time; nasopharyngeal temperature at circulatory arrest; combined with aortic bypass surgery; AMI; intraoperative transfusion of PRBCs; (IV) adjusted for age; gender; BMI; smoking history; aortic cross clamp time; nasopharyngeal temperature at circulatory arrest; combined with aortic bypass surgery; AMI; intraoperative transfusion of PRBCs; renal artery dissection or occlusion; Penn class; kidney malperfusion; preoperative shock; Bentall+TAR+FET. Conforming to the recommendations of the STROBE statement [39], the results were analyzed from unadjusted or minimally adjusted and fully adjusted in parallel. Whether the covariables was adjusted was determined according to the recommendations of the article published by The NEW ENGLAND JOURNAL of MEDICINE [16]: if, when the variable was added to this model, the matched odds ratio was changed by at least 10% then an adjustment was made. In addition, a generalized additive model (GAM) was also applied to identify linear relationships. The propensity score (PS) matching method was used to adjust intergroup differences between the non-AKI and AKI groups. We calculated the PS for each patient by matching variables (age; gender; BMI; diabetes mellitus; hypertension; smoking history; BUN; preoperative sCr; hemoglobin; hematocrit; eGFR). The balance in baseline covariant was assessed through paired t-tests and McNemar tests and standardized the mean differences as appropriate for categorical and continuous variables.

Interaction and stratified analysis were performed based on age (< 60 and ≥ 60 years), gender, BMI (< 24 kg/m^2^, 24-28 kg/m^2^, ≥28 kg/m^2^), hypertension, drinking history, smoking history, coronary artery disease, eGFR (< 60 mL/min/1.73m^2^, ≥60 mL/min/1.73m^2^), aortic cross clamp time (< 115 min, ≥115 min), circulatory arrest time (< 27 min, ≥27 min), hemoglobin (< 135 g/L, ≥135 g/L). All of the analysis was performed with the statistical software packages R (http://www.R-project.org, The R Foundation) and Empower Stats (http://www.empowerstats.com, X&Y Solutions, Inc., Boston, MA). A 2-sided significance level of 0.05 was regarded as statistically significant.

## Results

### Patients and inclusion characteristics

**Fig. 1:**
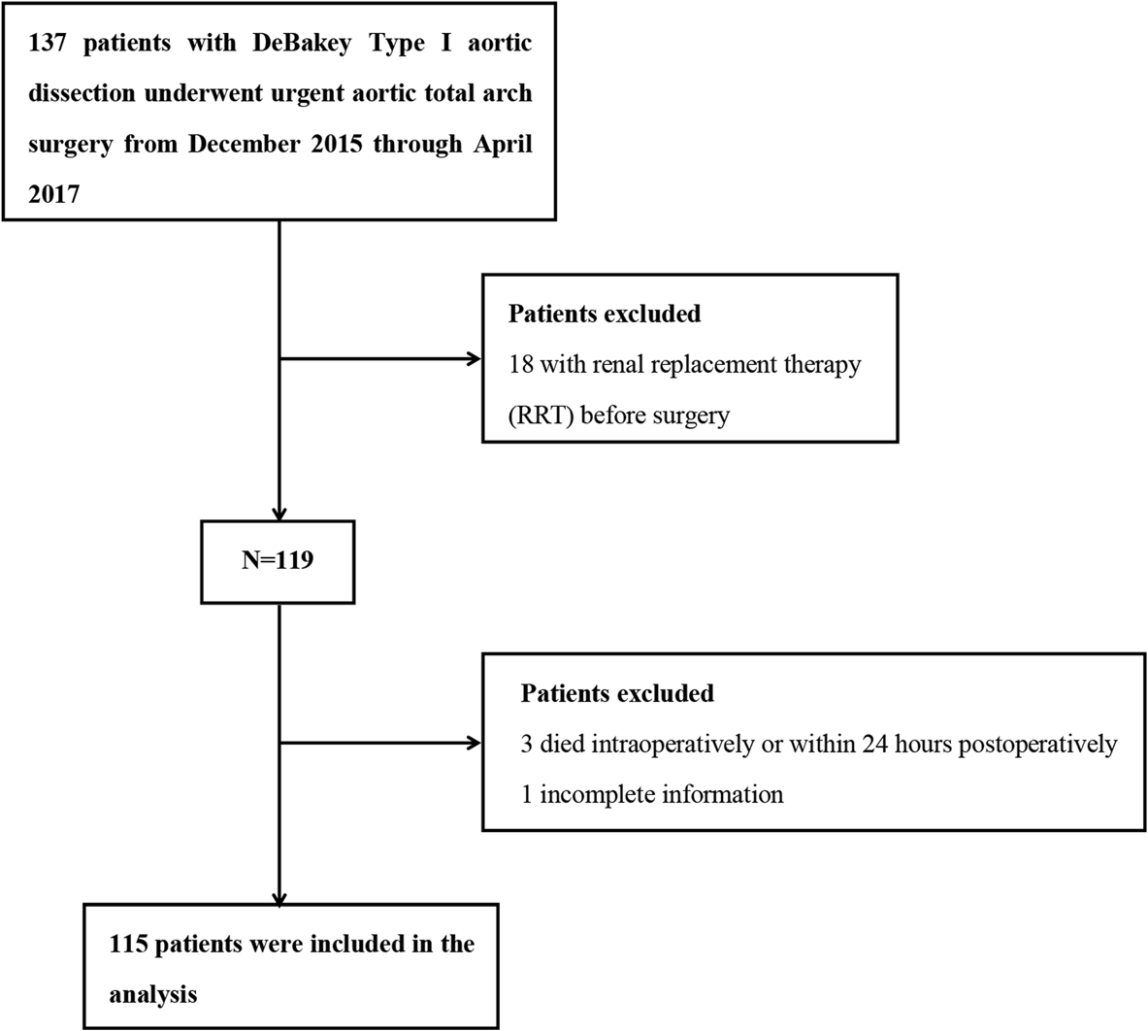
Flow diagram of the screening and enrollment of study patients

A total of 137 consecutive patients with ADTIAD who underwent emergent aortic TAR combined with an FET implant with CPB were included. All aortic TAR combined with a FET implant procedure, with or without aortic valve operation, were eligible. A total of 18 patients requiring RRT before surgery were excluded for the difficulty to evaluate the progression of renal dysfunction and three patients who died intraoperatively or within 24 h after operation were also excluded because no useful data were available to evaluate the AKI. One patient was excluded for incomplete information. As a consequence, a total of 115 consecutive persons were included in the eventual analysis. A flow chart of the screening and registration of study participants was given in Fig. 1.

### Characteristics of the study patients at baseline

After the exclusion criteria were used, 115 consecutive patients were admitted to this cohort. The average age was 47.8 ± 10.7 years. There were 86 (74.8%) male among these patients. The overall incidence of AKI was 53.0% (61 patients). The average CPB time was 211 ± 56 min. The average preoperative sCr was 86.2 ± 29.1umol/L, BUN was 7.2 ± 2.5 mmol/L. The average eGFR was 88.6 ± 22.6 mL/(min•1.73m^2^), Hemoglobin was 136.5 ± 17.3 g/L. The incidence of sepsis was 14.8%. A total of 23 patients required RRT. In-hospital mortality was 7.8%. The characteristics of the 115 patients at baseline who underwent thoracic aortic surgery for ADTIAD were shown in Table 1.

**Table 1:** Characteristics of the study patients at baseline

Variables	All patients (*n* = 115)
Age (year)	47.8 ± 10.7
Gender	
male	86 (74.8%)
female	29 (25.2%)
BMI (kg/m^2^)	26.2 ± 3.9
Diabetes mellitus	7 (6.1%)
Hypertension	92 (80.0%)
Previous cerebrovascular disease	6 (5.2%)
Coronary artery disease	6 (5.2%)
Smoking history	56 (48.7%)
Drinking history	23 (20.0%)
Hemopericardium	19 (16.5%)
BUN (mmol/L)	7.2 ± 2.5
Preoperative sCr (umol/L)	86.2 ± 29.1
eGFR mL/(min·1.73m^2^)	88.6 ± 22.6
Hemoglobin (g/L)	136.5 ± 17.3
Hematocrit (%)	39.4 ± 4.6
LVEF (%)	62.9 ± 6.0
Renal artery dissection or occlusion	17 (14.8%)
Penn class	
Class Aa	70 (60.9%)
Non class Aa	45 (39.1%)
Kidney malperfusion	7 (6.1%)
AMI	9 (7.8%)
Preoperative shock	19 (16.5%)
CPB time (min)	211 ± 56
Aortic cross clamp time (min)	123.8 ± 42.6
Circulatory arrest time (min)	27.4 ± 8.5
Bentall+TAR+FET	47 (40.9%)
Combined with CABG	8 (7.0%)
Combined with aortic bypass surgery	1 (0.9%)
Nasopharyngeal temperature (°C) at circulatory arrest	22.9 ± 1.6
Rectal temperature (°C) at circulatory arrest	25.3 ± 2.1
Intraoperative transfusion of PRBCs	73 (63.5%)
AKI	61 (53.0%)
Reoperation for bleeding	9 (7.8%)
Postoperative dialysis	23 (20.0%)
Sepsis	17 (14.8%)
Length of in hospital (day)	14.0 (10.0–18.0)
Length of ICU (day)	3.0 (1.0–6.2)
In-hospital mortality	9 (7.8%)

Results are expressed as n (%) or mean ± SD or median [IQR]

*AKI* acute kidney injury, *ADTIAD* acute DeBakey Type I aortic dissection, *AMI* acute myocardial infarction, *BMI* body mass index, *BUN* blood urea nitrogen, *CABG* coronary artery bypass grafting, *CPB* cardiopulmonary bypass, *eGFR* estimated glomerular filtration rate, *FET* frozen elephant trunk, *TAR* total arch replacement, *ICU* intensive care unit, *LVEF* left ventricular ejection fraction, *PRBCs* packed red blood cells, *sCr* serum creatinine, *SD* standard deviation, *IOR* interquartile range

### Univariate analysis of risk factors associated with postoperative acute kidney injury in patients with ADTIAD

The consequences of a univariate analyses were given in Table 2. These results revealed that BMI, eGFR and CPB time were all correlated with AKI. We also found smoking, drinking, preoperative hemoglobin levels, history of diabetes mellitus, hypertension, coronary artery disease, preoperative hematocrit, preoperative sCr, BUN, LVEF, renal artery dissection, Penn class, kidney malperfusion, coronary malperfusion, preoperative shock, aortic cross clamp time, circulatory arrest time and Bentall+TAR+FET were not associated with AKI.

**Table 2:** Univariate analysis of risk factors associated with postoperative AKI in patients with ADTIAD

Variable	Statistics	OR (95%CI)	*P*-value
Age (year)	47.8 ± 10.7	1.02 (0.98, 1.05)	0.339
Gender			
male	86 (74.78%)	1.0	
female	29 (25.22%)	1.35 (0.58, 3.17)	0.487
BMI (kg/m^2^)	**26.2 ± 3.9**	**1.18 (1.06, 1.33)**	**0.003**
Diabetes mellitus	7 (6.09%)	5.78 (0.67, 49.62)	0.110
Hypertension	92 (80.00%)	1.30 (0.52, 3.24)	0.576
Previous cerebrovascular disease	6 (5.22%)	0.42 (0.07, 2.41)	0.333
Coronary artery disease	6 (5.22%)	0.88 (0.17, 4.55)	0.878
Smoking history	56 (48.70%)	1.83 (0.87, 3.85)	0.110
Drinking history	23 (20.00%)	0.96 (0.38, 2.39)	0.926
Hemopericardium	19 (16.52%)	0.98 (0.37, 2.63)	0.969
BUN (mmol/L)	7.2 ± 2.5	1.03 (0.89, 1.19)	0.722
Preoperative sCr (umol/L)	86.2 ± 29.07	1.01 (1.00, 1.02)	0.147
eGFR mL/(min·1.73m^2^)	**88.6 ± 22.6**	**0.98 (0.96, 1.00)**	**0.013**
Hemoglobin (g/L)	136.5 ± 17.3	1.00 (0.97, 1.02)	0.686
Hematocrit (%)	39.4 ± 4.6	0.97 (0.89, 1.05)	0.440
LVEF (%)	62.9 ± 6.1	1.03 (0.97, 1.10)	0.354
Renal artery dissection or occlusion	17 (14.8%)	0.57 (0.20, 1.62)	0.292
Penn class			
Class Aa	70 (60.9%)	1.0	
Non class Aa	45 (39.1%)	1.18 (0.56, 2.50)	0.665
Kidney malperfusion	7 (6.1%)	1.19 (0.25, 5.59)	0.823
AMI	9 (7.8%)	3.37 (0.67, 16.98)	0.141
Preoperative shock	19 (16.5%)	0.98 (0.37, 2.63)	0.969
CPB time	**211 ± 56**	**1.09 (1.01, 1.17)**	**0.032**
Aortic cross clamp time (min)	123.8 ± 42.6	1.00 (0.99, 1.01)	0.401
Bentall+TAR+FET	47 (40.9%)	1.01 (0.48,2.13)	0.979
Combined with CABG	8 (7.0%)	1.52 (0.35, 6.67)	0.581
Combined with aortic bypass surgery	1 (0.9%)	^a^	0.992
Circulatory arrest time (min)	27.4 ± 8.5	1.00 (0.96, 1.05)	0.913
Nasopharyngeal temperature (°C) at circulatory arrest	22.9 ± 1.6	0.83 (0.65, 1.06)	0.143
Rectal temperature (°C) at circulatory arrest	25.3 ± 2.1	0.92 (0.77, 1.10)	0.356
Intraoperative transfusion of PRBCs	73 (63.5%)	0.90 (0.42, 1.92)	0.779
Reoperation for bleeding	9 (7.83%)	8.00 (0.97, 66.21)	0.054

Results are expressed as n (%) or mean ± SD or median [IQR]

*AKI* acute kidney injury, *ADTIAD* acute DeBakey Type I aortic dissection, *AMI* acute myocardial infarction, *BMI* body mass index, *BUN* blood urea nitrogen, *CABG* coronary artery bypass grafting, *CPB* cardiopulmonary bypass, *eGFR* estimated glomerular filtration rate, *FET* frozen elephant trunk, *TAR* total arch replacement, *LVEF* left ventricular ejection fraction, *PRBCs* packed red blood cells, *sCr* serum creatinine, *SD* standard deviation, *IOR* interquartile range

Bold value indicates significance at *p* < 0.05

^a^The result failed because of the small sample size

### The linear relationship between CPB time and AKI after adjusting for covariates

**Fig. 2:**
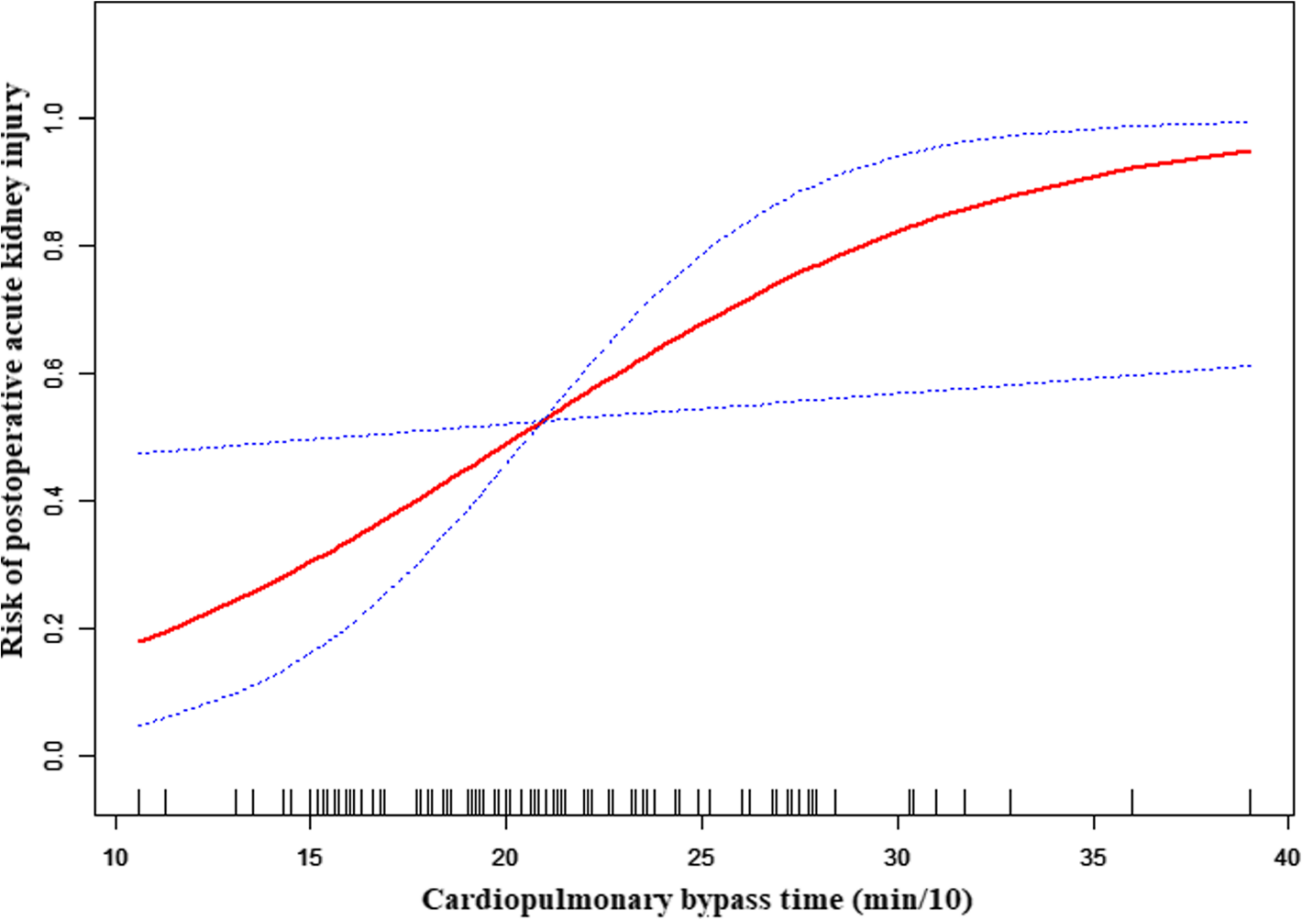
A linear relationship between CPB time and postoperative AKI was observed. Adjusting for adjusted for age; gender; BMI; smoking history; aortic cross clamp time; nasopharyngeal temperature at circulatory arrest; combined with aortic bypass surgery; AMI; intraoperative transfusion of PRBCs; renal artery dissection or occlusion; Penn class; kidney malperfusion; preoperative shock; Bentall+TAR+FET. In this figure, the red line indicates the estimated risk of acute kidney injury, and the dotted lines represent pointwise 95% CI

Spline smoothing was applied using GAM to explore the association between CPB time and AKI after adjusting for age; gender; BMI; smoking history; aortic cross clamp time; nasopharyngeal temperature at circulatory arrest; combined with aortic bypass surgery; AMI; intraoperative transfusion of PRBCs; renal artery dissection or occlusion; Penn class; kidney malperfusion; preoperative shock; Bentall+TAR+FET. A linear relationship between CPB time and AKI was shown in Fig. 2. The red points express the fitting spline. The black points express the 95% confidence intervals.

### Multivariable analysis to assess the independent impact of CPB time on postoperative AKI in patients with ADTIAD using non-adjusted and adjusted logistic regression model

Table 3 revealed the results of multivariate logistic regression analysis models for AKI based on different variable categories (preoperative, intraoperative and variables which are known to be related to AKI) included in each analysis. In adjusted model I, the result showed a significant association between CPB time and AKI [odds ratio (OR) =1.085; 95% confidence interval (CI): 1.007–1.170; *P* = 0.033]. In adjusted model II (adjusted age; gender), the result remained significant (OR = 1.092, 95% CI:1.012–1.179; *P* = 0.024). In adjusted model III (adjusted age; gender; BMI; smoking history; aortic cross clamp time; nasopharyngeal temperature at circulatory arrest; combined with aortic bypass surgery; AMI; intraoperative transfusion of PRBCs), the result remained significant (OR = 1.166, 95% CI:1.009–1.349; *P* = 0.031). In adjusted model IV (adjusted for age; gender; BMI; smoking history; aortic cross clamp time; nasopharyngeal temperature at circulatory arrest; combined with aortic bypass surgery; AMI; intraoperative transfusion of PRBCs; renal artery dissection or occlusion; Penn class; kidney malperfusion; preoperative shock; Bentall+TAR+FET.), the result still remained significant (OR = 1.171; 95% CI:1.002–1.368; *P* = 0.047).

**Table 3:** Multivariable analysis to assess the independent impact of CPB time on postoperative AKI in patients with ADTIAD using none adjusted and fully adjusted logistic regression model

Variable	Model I OR (95%CI)	*P-*value	Model II OR (95%CI)	*P-*value	Model III OR (95%CI)	*P-*value	Model IV OR (95%CI)	*P-*value
CPB time (per 10 min)	1.085 (1.007, 1.170)	0.033	1.092 (1.012, 1.179)	0.024	1.166 (1.009–1.349)	0.031	1.171 (1.002–1.368)	0.047

*AKI* acute kidney injury, *ADTIAD* acute DeBakey Type I aortic dissection, *AMI* acute myocardial infarction, *BMI* body mass index, *BUN* blood urea nitrogen, *CPB* cardiopulmonary bypass, *FET* frozen elephant trunk, *PRBCs* packed red blood cells, *sCr* serum creatinine, *TAR* total arch replacement, *OR* Odd Ratio, *95% CI* 95% confidence interval

Model I: adjust for none

Model II: adjust for age; gender

Model III: adjust for age; gender; BMI; smoking history; aortic cross clamp time; nasopharyngeal temperature at circulatory arrest; combined with aortic bypass surgery; AMI; Intraoperative transfusion of PRBCs

Model IV: adjust for age; gender; BMI; smoking history; aortic cross clamp time; nasopharyngeal temperature at circulatory arrest; combined with aortic bypass surgery; AMI; Intraoperative transfusion of PRBCs; renal artery dissection or occlusion; Penn class; kidney malperfusion; preoperative shock; Bentall+TAR+FET

### Sensitivity analysis using PS matching

To reduce the influence of confounding variables, we subsequently utilized the derived PS values to match 61 patients in the AKI group with the patients in the non-AKI group at a ratio of 1:1 using a greedy matching algorithm [4]. Finally, 64 patients were successfully matched, 32 patients with AKI and 32 without AKI. After all PS matches were performed, all variables were shown in Additional file 2: Table S1. The PS matching map was shown in Additional file 1: Figure S1. After PS matching (*n* = 64 pairs), the association between CPB time and AKI was still statistic significant (OR = 1.128, 95% CI:1.004–1.267; *P* = 0.043). The results were shown in Additional file 3: Table S2.

### Stratified analysis

**Fig. 3:**
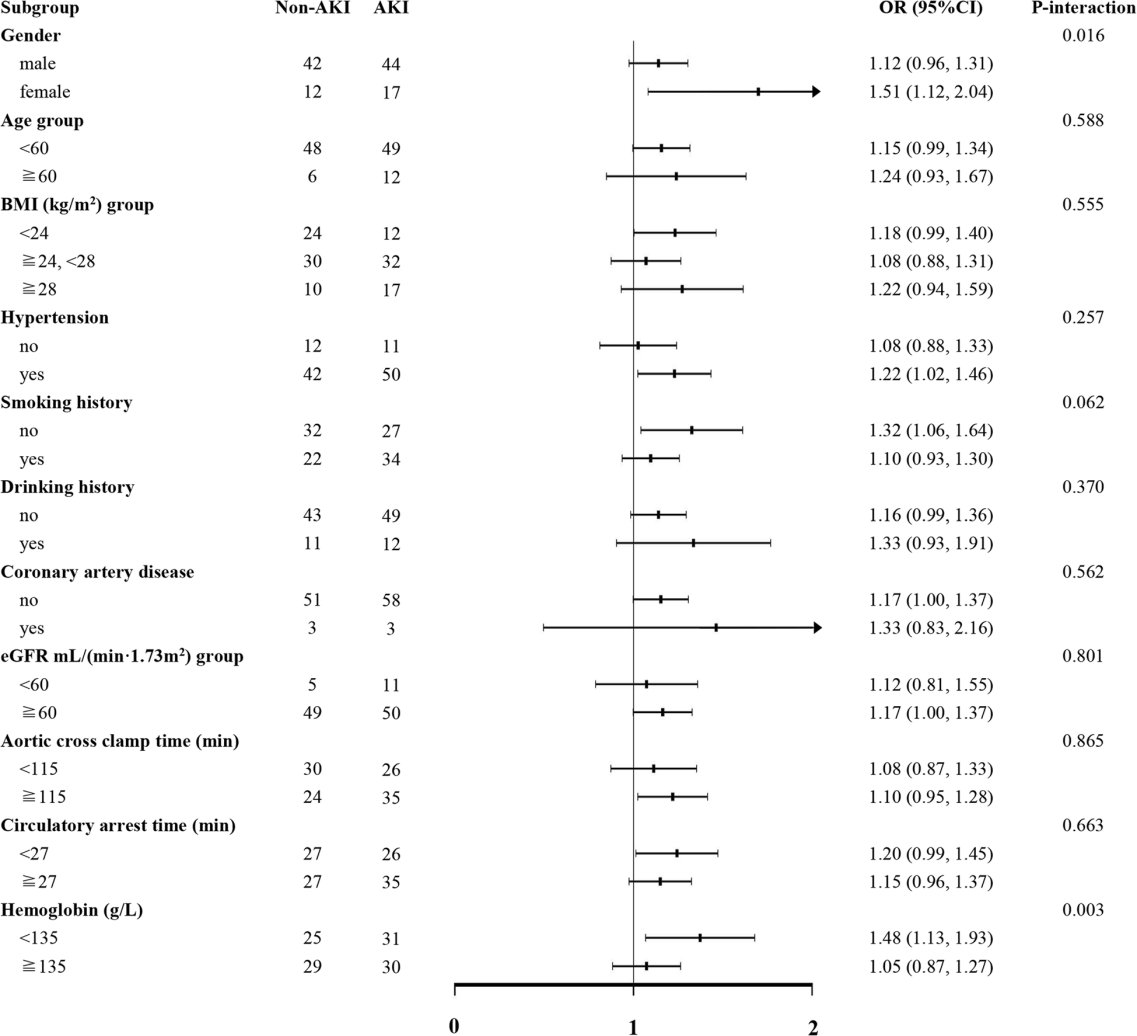
Subgroup analysis of the association between CPB time and AKI in patients with ADTIAD. Each stratification adjusted for all the factors: (adjusted for age; gender; BMI; smoking history; aortic cross clamp time; nasopharyngeal temperature at circulatory arrest; combined with aortic bypass surgery; AMI; intraoperative transfusion of PRBCs; renal artery dissection or occlusion; Penn class; kidney malperfusion; preoperative shock; Bentall+TAR+FET.) except the stratification factor itself

Stratified analysis was performed in patients with age (< 60 and ≥ 60 years), gender, BMI (< 24 kg/m^2^, 24-28 kg/m^2^, ≥28 kg/m^2^), hypertension, smoking history, drinking history, coronary artery disease, eGFR (< 60 mL/min/1.73m^2^, ≥60 mL/min/1.73m^2^), aortic cross clamp time (< 115 min, ≥115 min), circulatory arrest time (< 27 min, ≥27 min), hemoglobin (< 135 g/L, ≥135 g/L). CPB time was still an independent predictor of post-operation AKI in one of these high-risk subgroups. And, there was no interaction with AKI among these groups. Stratified analysis was given in Fig. 3.

## Discussion

In this retrospective cohort study of 115 patients who underwent emergent thoracic aortic surgery for ADTIAD, we found that the CPB time was an independent predictor of postoperative AKI (OR = 1.171; 95% CI:1.002–1.368; *P* = 0.047). A 10 min increase in CPB time was associated with a 17.1% higher risk of postoperative AKI. After PS matching was applied to adjust for the potential confounding factors, the results still remained statistic significant. Further confirming the relationship between CPB time and postoperative AKI and validates our hypothesis. Thus, we should improve surgical techniques in surgery as soon as possible to reduce CPB time.

There have been several studies on the risks of postoperative AKI in patients underwent aortic surgery. Mori Y et al. [28] analyzed 135 patients who underwent aortic arch surgery under deep hypothermic circulatory arrest (DHCA). They found preoperative hypertension, emergency surgery and DHCA were associated with postoperative AKI. There were no differences in other characteristics, such as age, gender, BMI, duration of CPB time and aortic cross clamping time. The reason may be that the study population was different. A retrospective study included 737 patients who underwent aortic surgery with CPB by Kim WH et al. [17] they found the following variables were significantly associated with AKI after adjustment for other risk factors: age older than 60 years (OR = 1.83, 95% CI:1.13–2.96, *P* = 0.015), preoperative GFR < 60 mL/min•1.73m^2^ (OR = 2.36, 95% CI:1.40–3.96, *P* = 0.001), preoperative LVEF< 55% (OR = 2.08, 95% CI:1.14–3.79, *P* = 0.017), operation time > 7 h (OR = 2.63, 95% CI:1.63–4.24, *P* < 0.001) and intraoperative oliguria (< 0.5 mL/kg/h) (OR = 2.81, 95% CI: 1.37–5.77, *P* = 0.005) or intraoperative furosemide use (OR = 1.99, 95% CI:1.25–3.16, *P* = 0.004). CPB time was not found to be association with postoperative AKI. The reason might be that they did not include the CPB time in the multivariate logistic regression equation although the baseline data of CPB time between non-AKI group and AKI group was different. Another study conducted by Kowalik MM et al. [19, 20], they reported that CPB time did not influence the development of postoperative AKI. These controversial results might be attributed to confounding factors in heterogeneous patient cohorts.

This result was consistent with what Englberger L et al. identified [22], they studied 851 patients who underwent elective thoracic aortic operation with and without DHCA, and postoperative AKI was defined by consensus RIFLE (Risk, Injury, Failure, Loss of function, End-stage renal disease) criteria. They found longer CPB time (per 10 min) was a significantly risk factor of postoperative AKI (OR = 1.09, 95% CI:1.05–1.12; P = 0.001). Roh GU et al. [32] analyzed 98 patients underwent graft replacement of the thoracic aorta in patients with aortic dissection, and they found long CPB duration (> 180 min; OR = 7.50; *P* = 0.008) was an independent risk factor for postoperative AKI which was consistent with our study. Geoge J et al. [3] analyzed 586 patients who undergoing elective aortic hemiarch reconstruction, they also found CPB time (min) (OR = 1.01, 95% CI: 1.00–1.01; *P* = 0.03) was an independent risk factor of postoperative AKI. Moreover, several other studies [5, 13, 18, 23, 30] had found that CPB time was an independent predictor of postoperative AKI in patients underwent cardiac and vascular surgery which further verified our findings.

The potential mechanism for this association between CPB time and AKI was unclear. Mamikonian et al. [27] investigated the association between hemolysis and postoperative AKI in 42 children who undergoing cardiac surgery with CPB. They found that significant hemolysis occurred during CPB and was related to the development of postoperative AKI. Decreasing CPB-induced hemolysis or attenuating the effects of CPB-induced hemolysis by augmenting endogenous mechanisms that exist to scavenge and remove free hemoglobin may provide a way to more rapidly clear excess plasma free hemoglobin, decrease oxidant injury, and minimize the toxic effects of acute hemolysis and reduce the incidence of AKI. L. Lannemyr et al. [25] performed a detailed analysis on the association between the renal tubular injury and CPB. They found that a renal tubular cell injury was detected early after onset of CPB and with a peak biomarker increased early after the CPB during cardiac surgery. The magnitude of renal tubular injury was independently association with CPB duration and the degree of rewarming. The means to decrease the risk for tubular injury were decreasing the CPB duration and avoiding hypothermia.

The incidence of AKI after operation in our study was similar to that found in two former studies that used RIFLE criteria [1, 2, 14]. Hobson et al. [14] documented an incidence of AKI following aortic surgery of 55%, which was comparable to our finds. Although there were little data concerning the patients’ characteristics in that study, their study cohort was seemingly heterogeneous according to diagnosis and surgical condition. Another study recorded an AKI incidence rate of 48% in 267 patients following aortic arch surgery with DHCA, including 36% of AAD and 36% of emergency operations [1, 2]. However, Englberger et al. [10] recently showed a relatively low incidence rate of AKI (17.7%) and RRT (2.1%) among 851 patients who underwent elective thoracic aortic surgery with and without DHCA. In this study, both acute dissection and emergency surgery, which had been known independent predictors for postoperative AKI [12], were excluded. Considering that our study cohort underwent emergent aortic TAR combined with a FET implant as a result of ADTIAD, the incidence of AKI (53%) was not surprising.

This study has several strengths. First, the patients selected for this study comprised a homogeneous population of patients with ADTIAD underwent urgent thoracic aortic surgery with moderate hypothermic circulatory arrest (HCA). Second, because the worst sCr level was available daily for all patients during the entire study period, AKI development could be assessed for all patients accurately. Finally, we adopted the KDIGO guidelines for AKI instead of the previous two classifications since the KDIGO guidelines have been revised most recently and offered clarity and simplicity in clinical use. Increased understanding of the predictors associated with AKI development is significant, and highlighting CPB as a risk factor may guide clinical management strategies intraoperatively which included improving surgical skills, operating as soon as possible and reducing CPB time.

### Study limitations

Our research also has several limitations which are worth noting. First, it is a retrospective cohort study, and for this purpose, can constitute an association, but not a causality between CPB time and AKI. Second, aortic TAR combined with a FET implant is a preferred choice to treat ADTIAD at our center, while other centers may select more conventional procedures, this may result in a discrepancy in the outcomes among different studies, but our study cohort also forms a homogenous population who had undergone this procedure, which adds internal validity. Finally, nearly all patients were male in our cohort. Caution is required when extrapolating these findings to female patients.

## Conclusions

CPB time is an independent predictor of postoperative AKI in patients underwent thoracic aortic surgery for ADTIAD. Further understanding of the molecular mechanism of this association is crucial to the design of preventative therapies.

## Additional files

Additional file 1: **Figure S1.** PS matching method was used to adjust intergroup differences between AKI and non-AKI group. We calculated the PS for each patient by matching variable (age; gender; BMI; diabetes mellitus; hypertension; smoking history; BUN; preoperative sCr; hemoglobin; hematocrit; eGFR). (TIF 23 kb)

Additional file 2: **Table S1.** Characteristics of the study patients at baseline after propensity score matching. (DOC 40 kb)

Additional file 3: **Table S2.** Multivariable analysis to assess the independent impact of CPB time on postoperative AKI in patients with ADTIAD after PSM. (DOC 33 kb)

## Abbreviations

ACT: Activated clotting time
ADTIAD: Acute DeBakey Type I aortic dissection
AKI: Acute kidney injury
AKIN: Acute Kidney Injury Network
AMI: Acute myocardial infarction
BMI: Body mass index
BUN: Blood urea nitrogen
CABG: Coronary artery bypass grafting
CAG: Coronary angiography
CI: Confidence interval
CPB: Cardiopulmonary bypass
DHCA: Deep hypothermic circulatory arrest
GAM: Generalized additive model
HCA: Hypothermic circulatory arrest
ICU: Intensive care unit
KDIGO: Kidney Disease Improving Global Outcomes
LVEF: Left ventricular ejection fraction
OR: Odds ratio
PRBCs: Packed red blood cells
PS: Propensity score
RIFLE: Risk, Injury, Failure, Loss and End-stage
RRT: Eenal replacement therapy
SCP: Selective cerebral perfusion
sCr: Serum creatinine
SD: Standard deviation
