# Supplementary figures and images for: Cardiopulmonary bypass time is an independent risk factor for acute kidney injury in emergent thoracic aortic surgery: a retrospective cohort study
Source: J Cardiothorac Surg. 2019 May 7;14:90. doi: 10.1186/s13019-019-0907-x (PMC6505293; doi:10.1186/s13019-019-0907-x)

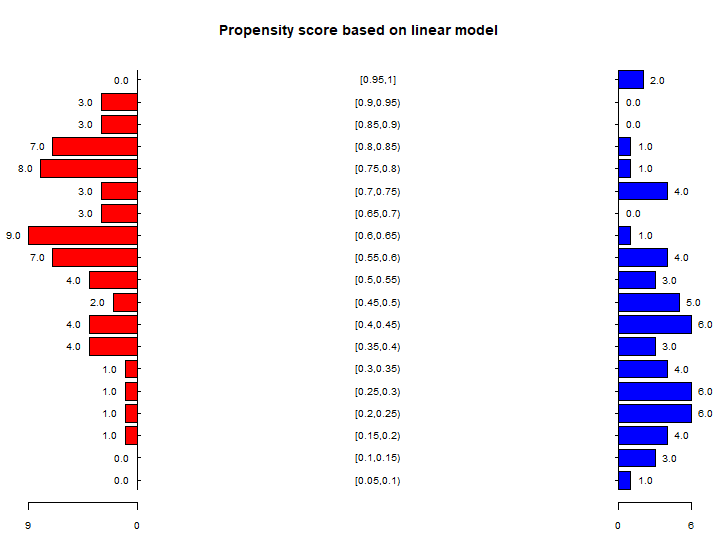

Supplement: Supplementary file 1 — Figure S1. PS matching method was used to adjust intergroup differences between AKI and non-AKI group. We calculated the PS for each patient by matching variable (age; gender; BMI; diabetes mellitus; hypertension; smoking history; BUN; preoperative sCr; hemoglobin; hematocrit; eGFR). (TIF 23 kb) [file 13019_2019_907_MOESM1_ESM.tif]
