# Supplementary material for: Cardiopulmonary bypass time is an independent risk factor for acute kidney injury in emergent thoracic aortic surgery: a retrospective cohort study
Source: J Cardiothorac Surg. 2019 May 7;14:90. doi: 10.1186/s13019-019-0907-x (PMC6505293; doi:10.1186/s13019-019-0907-x)
Supplement: Supplementary file 2 — Table S1. Characteristics of the study patients at baseline after propensity score matching. (DOC 40 kb) [file 13019_2019_907_MOESM2_ESM.doc]

**Supplement Table 1:** Characteristics of the study patients at baseline after propensity score matching.

| **Variables** | **Non-AKI (n=32)** | **AKI (n=32)** | ***P* -value** |
| --- | --- | --- | --- |
| Age | 49. ± 11.8 | 47.9 ± 10.2 | 0.693 |
| Gender |  |  | 0.773 |
| male | 23 (71.9%) | 25 (78.1%) |  |
| female | 9 (28.1%) | 7 (21.9%) |  |
| BMI (kg/m2) | 26.5 ± 3.4 | 26.5 ± 3.4 | 0.963 |
| Diabetes mellitus | 1 (3.1%) | 2 (6.2%) | 1.000 |
| Hypertension | 27 (84.4%) | 28 (87.5%) | 1.000 |
| Smoking history | 18 (56.2%) | 18 (56.2%) | 1.000 |
| BUN (mmol/L) | 7.2 ± 2.2 | 7.0 ± 2.1 | 0.657 |
| Preoperative sCr (umol/L) | 84.5 ± 32.1 | 84.1 ± 26.8 | 0.956 |
| eGFR mL/(min·1.73㎡) | 89.4 ± 20.9 | 90.5 ± 22.4 | 0.851 |
| Hemoglobin (g/L) | 137.0 ± 20.2 | 138.4 ± 16.4 | 0.761 |
| Hematocrit (%) | 39.4 ± 5.6 | 39.6 ± 4.4 | 0.889 |
| CPB time (min/10) | 19.29 ± 4.46 | 22.54 ± 6.45 | 0.022 |

AKI = acute kidney injury; BMI = body mass index; eGFR = estimated glomerular filtration rate

For continuous variables: (N) Mean ± SD, Standardized difference = abs (Mean1-Mean0)/sqrt((S1±S2)/2)
For categorical variables: N (%), Standardized difference = abs(P1-P0)/sqrt((P1*(1-P1) ±P0*(1-P0))/2)

Matching variable：Age; Gender; BMI; Diabetes mellitus; Hypertension; Smoking history; BUN; Preoperative sCr; Hemoglobin; Hematocrit; eGFR.
