# Supplementary material for: Cardiopulmonary bypass time is an independent risk factor for acute kidney injury in emergent thoracic aortic surgery: a retrospective cohort study
Source: J Cardiothorac Surg. 2019 May 7;14:90. doi: 10.1186/s13019-019-0907-x (PMC6505293; doi:10.1186/s13019-019-0907-x)
Supplement: Supplementary file 3 — Table S2. Multivariable analysis to assess the independent impact of CPB time on postoperative AKI in patients with ADTIAD after PSM. (DOC 33 kb) [file 13019_2019_907_MOESM3_ESM.doc]

**Supplement Table 2:** Multivariable analysis to assess the independent impact of CPB time on postoperative AKI in patients with ADTIAD after PSM.

| **Variable** | **PSM** OR (95%CI) | ***P* -value** |
| --- | --- | --- |
| CPB time (per 10 min) | 1.128 (1.004, 1.267) | 0.043 |

AKI = acute kidney injury；ADTIAD = acute DeBakey Type I aortic dissection; BMI = body mass index;

CPB = cardiopulmonary bypass；OR = Odd Ratio；95% CI = 95% confidence interval;

PSM = propensity score matching;

Matching variable: Age; Sex; BMI; Diabetes mellitus; Hypertension; moking history; BUN; Preoperative sCr; Hemoglobin; Hematocrit; eGFR;

adjust for: PP. SCORE
